# Sir Gordon Morgan Holmes (1876–1965): one of the founders of modern neurology

**DOI:** 10.1007/s10072-017-3180-6

**Published:** 2017-11-07

**Authors:** Jarosław Jerzy Sak, Andrzej Grzybowski, Jacek Baj

**Affiliations:** 1 Department of Ethics and Human Philosophy, Medical University of Lublin, ul. Staszica 4/6 (Collegium Maximum), 20-081 Lublin, Poland; 2 Department of Ophthalmology, Poznań City Hospital, ul. Szwajcarska 3, 61-285 Poznań, Poland; 3 Department of Ophthalmology, University of Warmia and Mazury, Olsztyn, Poland; 4 Department of Normal Anatomy, Medical University of Lublin, ul. Jaczewskiego 4 (Collegium Anatomicum), 20-090 Lublin, Poland

**Keywords:** Gordon Morgan Holmes, Stewart-Holmes maneuver, Stewart-Holmes test, Gordon-Holmes syndrome, Holmes-Adie pupil

## Abstract

Sir Gordon Morgan Holmes (1876–1965) was one of the most important founders of modern neurology and a great teacher and scientist. He was the first scientist to challenge the theory of the unitary function of the cerebellum and described cerebellar disorders. Holmes together with Thomas Grainger Stewart (1877–1957) described 40 cases of the rebound phenomenon in cerebellar disease (Stewart-Holmes maneuver or Stewart-Holmes test). He also described the symptoms of inherited neurodegenerative spinocerebellar ataxia involving the olivary nucleus (Gordon-Holmes syndrome). Independently from the Australian neurologist William John Adie (1886–1935), he described the partial iridoplegia (Holmes-Adie pupil or Holmes-Adie syndrome). His teaching skills became clearly visible in Goulstonian and Croonian lectures dedicated to spinal cord injuries.

## Introduction

**Fig. 1:**
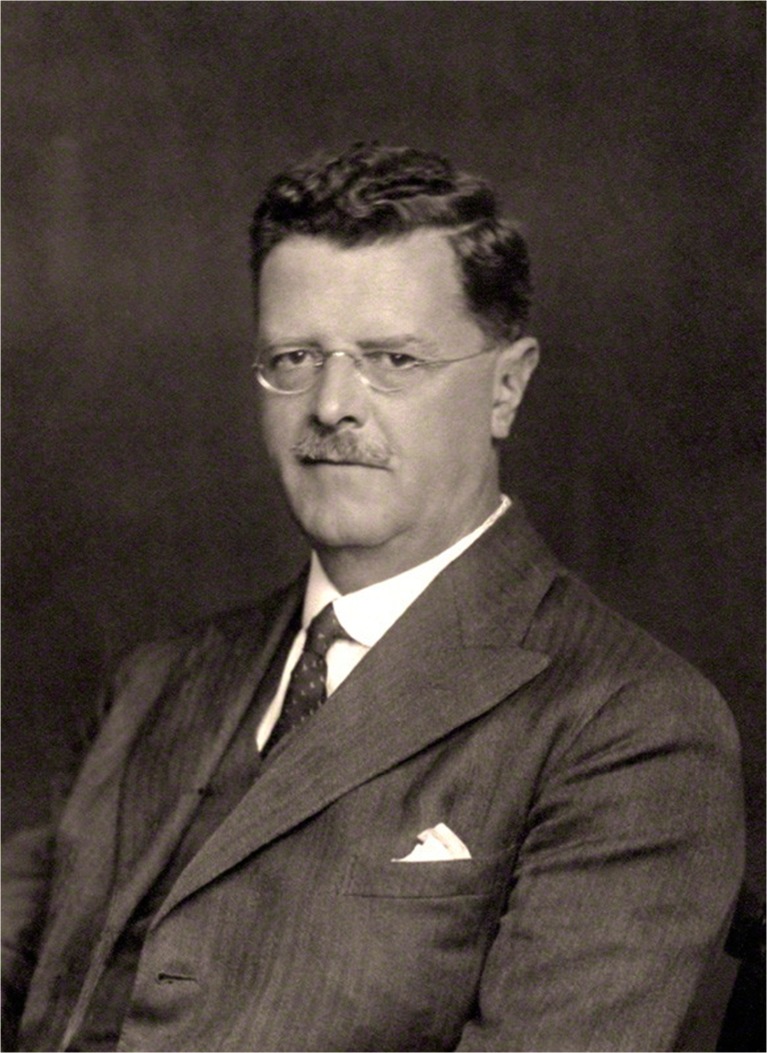
Sir Gordon Morgan Holmes (1876–1965). Photo made by Stoneman W, 16 June 1933. Source: National Portrait Gallery, London. http://www.npg.org.uk/collections/search/portraitLarge/mw109878/Sir-Gordon-Morgan-Holmes

2016 marks the 140th anniversary of the birth of an Irish neurologist Sir Gordon Morgan Holmes (1876–1965) (Fig. 1) who through his research on the cerebellum and the visual cortex made a significant contribution to the development of neurology. He is also considered one of the most important founders of modern neurology and a great teacher and scientist [1, 2]. Nowadays, neurologists use eponyms such as Gordon-Holmes syndrome and Holmes-Adie syndrome in symptomatologic descriptions. What is more, the Stewart-Holmes test is still an important part of neurological diagnostics.

## Biographical sketch

Holmes was born on January 22, 1876, in Dillon House, Castlebellingham (County Louth, Ireland) 40 miles north of Dublin. He had three brothers and three sisters. His father Gordon Holmes was a farmer from the Louth County. Holmes’ choice of a medical career was probably influenced by his mother’s Kathleen’s passing away when he was just a child [2, 3]. The first stage of his education was at the Dundalk Educational Institute where despite his struggle with dyslexia, he was an excellent pupil [3]. He graduated from his medical studies at Trinity College in Dublin in 1897, obtaining a B.A., Senior Moderator in Natural Science degree. In the beginning of his career, he worked as a ship’s surgeon on a ship sailing to New Zealand. After returning from the Antipodes a year later, thanks to the Stewart Scholarship in Nervous and Mental Diseases from Trinity College, he was able to participate in a foreign scientific internship at the Senckenberg Institute in Frankfurt-am-Main (Germany) [2, 3]. He gained his knowledge in comparative anatomy and histology of the nervous system under the direction of Carl Weigert (1845–1904) and in neuroanatomy under Ludwig Edinger (1855–1918) [1–3]. Edinger, appreciating the abilities of the young doctor, offered him a job as an assistant [1]. However, in 1903, Holmes decided to return to the British Isles and tie his professional career for subsequent years with the National Hospital for Nervous Diseases, Queen Square in London, which apart from the Pitié-Salpêtrière Hospital in Paris, was one of the two most important European neurological centers of that time [3]. Initially, he worked there as a house-physician and then as a Resident Medical Officer [1–3]. In 1903, Holmes also obtained his MD degree. In 1906, he became the director of clinical research, and in 1909, was appointed honorary physician to the National Hospital. This position allowed him to develop a private neurological practice on Harley Street in London. During this time, he also served as a consulting physician to the Charing Cross Hospital and the Royal Ophthalmic Hospital (Moorfields Eye Hospital) [3]. Since 1909, he carried out intensive research in neuroanatomy and neuropathology and since 1911 also in the field of neurophysiology. In 1910, he was going to join an expedition to the South Pole organized by Robert Falcon Scott (1868–1912); however; these plans were ruined due to a ruptured Achilles tendon [3]. Scott’s expedition reached the South Pole in 1912, but the way back turned out tragic for all of the participants. Each year, Holmes organized a three-day boat trips on the Thames from Oxford to London for his co-workers and students. After the outbreak of the First World War in 1914, Holmes became the Consulting Neurologist to the Royal Army Medical Corps in France. Together with a neurosurgeon Percy Sargent (1873–1933), they organized a hospital in France for treating head wounds. For his activities during the WWI, Holmes received the Commander of the Most Excellent Order of the British Empire (CBE) and the Order of St Michael and St George in class Companion (CMG). After the war, in 1918, he married Rosalie Jobson, an Oxford graduate and an international sportswoman, with whom he had three daughters: Kathleen, Rosalie, and Elizabeth. The family lived at Wimpole Street 9 in London. In the period between 1922 and 1937, Holmes served as the Editor-in-Chief of the journal “Brain”. He held weekly, extremely popular, analysis of neurological cases in the National Hospital at Queen Square for physicians in training [1–3]. He was a co-founder of the Association of British neurologists (ABN) and the constituent meeting was held on 28 July 1932 at his home in London. A year later, in recognition of his achievements, he was elected a Fellow of the Royal Society (F.R.S.) [3]. He was repeatedly invited to opening ceremonies of new neurological hospitals. During one such ceremony—the gala opening of the Montreal Neurological Institute in 1934, he quoted the words of Francis Bacon (1561–1626) as a motto for neurology: “Desire to seek, patience to doubt, fondness to meditate, slowness to assert, readiness to reconsider, carefulness to dispose and set in order” [2]. During the Second International Congress of Neurology held in 1935 in London, he served as President of Congress and in the years between 1936 and 1938. After the outbreak of the Second World War, he continued his work and his scientific and didactic activity in the Charing Cross Hospital and held a position as a consultant in the field of neurology at the Emergency Medical Service. Because of their house being partially destroyed by the bombing, Holmes had to leave his house of Wimpole Street and move to a country house in Farnham (Surrey County, South East England). There, after his retirement, he indulged in his passions which were golfing and cultivating his garden. He received numerous honorary degrees (DSc, Dublin in 1933; DSc, National University of Ireland in 1941; the DCL, Durham in 1944; the LLD, Edinburgh in 1952) and was knighted in 1951. He died in Farnham on 29 December 1965 at the age of 89 [1–3].

## Holmes’ scientific achievements

Prior to the outbreak of the WWI Holmes, together with Henry Head (1861–1940) worked on the identification of visual pathways [4]. He was a careful researcher who during the WWI made numerous observations on the dysfunctions of the cerebellum, spinal cord, and visual impairments among patients with gunshot wounds [5]. It should be underlined that the development of military technology during the WWI indirectly contributed to the advancement of knowledge about pathophysiology of the occipital cortex and the visual pathways. The bullets fired from rifles with low caliber but high kinetic energy relatively easily penetrated the skull without making cavitation or shock waves in the brain and neither the US marine Borps Doughboy helmet nor the British “Brodie” helmets were good protection for the occipital part of the soldiers’ heads [6, 7]. Skull fractures and occipital cortex damage were quite frequent on the WWI battlefields due to gunshot wounds. Therefore, Holmes as well as other physicians had numerous opportunities to conduct observations and analyzes, which in turn significantly contributed to the progress of understanding of the visual pathways [5–7]. Holmes in collaboration with the Ophthalmic Surgeon Consulting William Lister (1868–1944) performed perimetry on over 400 wounded soldiers. He mapped the line trajectory of gunshot wounds using X-rays and cross-sectional models of skull and brain [7]. He included his observations in among others the Goulstonian Lectures [8]. In 1918, Holmes described six cases of a visual disorientation with optic ataxia and bilateral posterior parietal cortex lesions which were due to perforating gunshot injuries of the head (Bálint-Holmes syndrome) [9].

He was also the first scientist to challenge the theory of the unitary function of the cerebellum and described cerebellar disorders through the following symptoms ataxia, asthenia, adiadochokinesia, and rebound [10]. Holmes together with Thomas Grainger Stewart (1877–1957) [1] described and explained 40 cases of the rebound phenomenon in cerebellar disease (Stewart-Holmes maneuver or Stewart-Holmes test) [11]. Holmes also described the symptoms of inherited neurodegenerative spinocerebellar ataxia involving the olivary nucleus (Gordon-Holmes syndrome). In 1931, independently from the Australian neurologist William John Adie (1886–1935), with whom he worked during his stay in France, he described the partial iridoplegia (Holmes-Adie pupil or Holmes-Adie syndrome) [12].

## Holmes’ impact on contemporary neurology

It is worth to underline that Holmes’ achievements in mapping the visual pathways have had a decisive impact on the progress of neurological knowledge although many other researchers including Pierre Marie (1853–1940), Walthur Poppelreuter (1886–1939), George Riddoch (1888–1947), or Sir Charles Symonds (1890–1978) conducted similar research in this field [6, 7]. Holmes accurately identified the main features of the retinal projection on the cortex. The method of testing the visual pathways used by Holmes in the WWI was also applied during similar research conducted in wounded soldiers of the WWII [6]. Holmes also contributed to the increase of knowledge of cerebellar disorders. The Stewart-Holmes test permanently entered the arsenal of neurological disorders diagnostic methods. Essential for modern neurological diagnostics is the Holmes-Adie syndrome, which is differentiated from the generalized neuropathy [13]. What is more, recent research suggests a genetic predisposition of the Gordon-Holmes syndrome [14].
